# Prosthetic Rehabilitation in Marble Bone Disease

**DOI:** 10.5005/jp-journals-10005-1079i

**Published:** 2010-09-15

**Authors:** Usha Rehani, Vivek K Adlakha, Preetika Chandna, Abhay Agarwal, Vivek Rana, Pooja Malik

**Affiliations:** 1 Professor and Head, Department of Pedodontics and Preventive Dentistry, Subharti Dental College, Meerut Uttar Pradesh, India; 2 Lecturer, Department of Pedodontics and Preventive Dentistry, Subharti Dental College, Meerut, Uttar Pradesh, India; 3 Professor, Department of Pedodontics and Preventive Dentistry, Subharti Dental College, Meerut, Uttar Pradesh, India; 4 Reader, Department of Pedodontics and Preventive Dentistry, Subharti Dental College, Meerut, Uttar Pradesh, India; 5 Postgraduate Student, Department of Pedodontics and Preventive Dentistry, Subharti Dental College, Meerut, Uttar Pradesh, India

**Keywords:** Osteopetrosis, Osteomyelitis, Marble bone disease.

## Abstract

Osteopetrosis is a rare metabolic disease.^1^ Dental abnormalities may be attributed to the pathological changes in osteopetrosis. Patients with osteopetrosis are especially susceptible to osteomyelitis of mandible.^1^ A 13-year-old girl presented with complaint of jaw swelling on left side. The radiograph of left mandible (lateral oblique view) showed small sequestrum with irregularity and erosions of the man-dibular cortical margins. This case report aims at diagnosis and treatment of osteopetrosis with superadded mandibular osteomyelitis―a are entity.

## INTRODUCTION

Marble bone disease (Osteopetrosis; Osteosclerosis Fragilis Generalisata) is an inherited, rare autosomal bone disorder of unknown etiology. It was first described in 1904 by Albers-Schonberg,^2^ hence the disease also gets a name Albers-Schonberg disease. This disorder includes impaired osteoclast function and marked increase in bone density.

The estimated prevalence of osteopetrosis is 1 in 100,000-500,000. It takes 2 major clinical forms-the autosomal dominant adult (benign) form is associated with few or no symptoms and the autosomal recessive infantile (malignant) form, if untreated, is typically fatal during infancy or early childhood.

A rarer autosomal recessive (intermediate) form presents during childhood with some signs and symptoms of malignant osteopetrosis. In especially rare cases, osteo-petrosis may exist as lethal, transient infantile and postinfectious forms. Most children born with the malignant form of osteopetrosis die during infancy. Due to better medical care the life expectancy of these patients has increased in recent years.

Most studies of osteopetrosis have concentrated on medical aspects (hepatosplenomegaly, anemia, increased susceptibility to infections and the most common is respiratory tract infections, cardiac disorders, multiple fractures, etc).^3^ With increasing age, however, dental development and tooth eruption become a practical and medical problem.^4^ In these patients to improve oral hygiene, especially in areas of exposed mandibular bone, 0.2% chlorhexidine formulations can be used. Fluoride applications can be done to decrease the susceptibility to dental caries.

This paper discusses a rare case of osteopetrosis with mandibular osteomyelitis with the purpose to review the entity and reemphasize an important, less obvious, clinical presentation of osteopetrosis with mandibular osteomyelitis.

## CASE REPORT

**Fig. 1:**
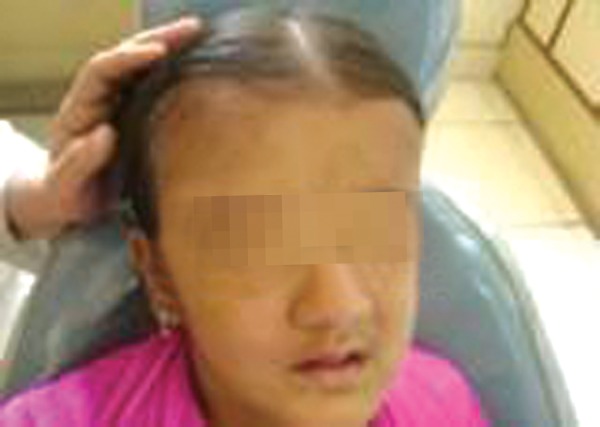
Extraoral view of a patient with osteopetrosis

**Fig. 2:**
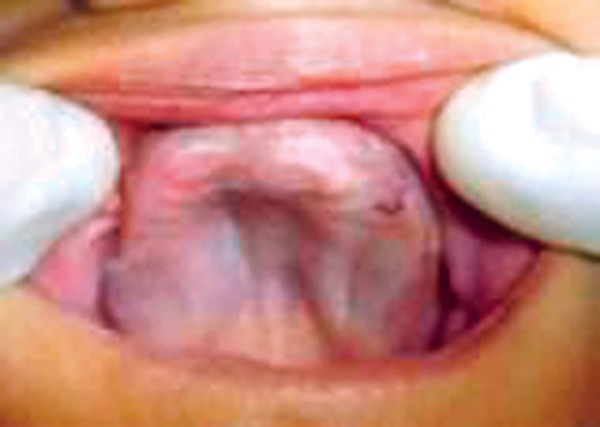
Maxillary arch (intraoral view)

**Fig. 3:**
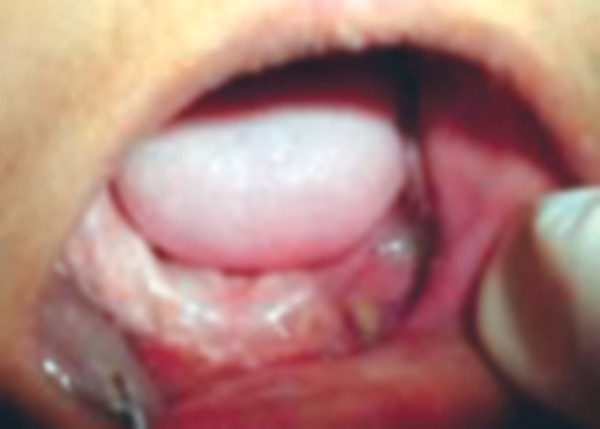
Mandibular arch (intraoral view)

A 13-year-old girl reported to the Department of Pedodontics and Preventive Dentistry, Subharti Dental College, Meerut with chief complaint of missing teeth (Fig. 1). A detailed history was taken, the patient had loss of vision and hearing impairment. Intraoral examination showed pale oral mucosa, small sequestrum on left side of the mandible (Figs 2 and 3).

**Fig. 4:**
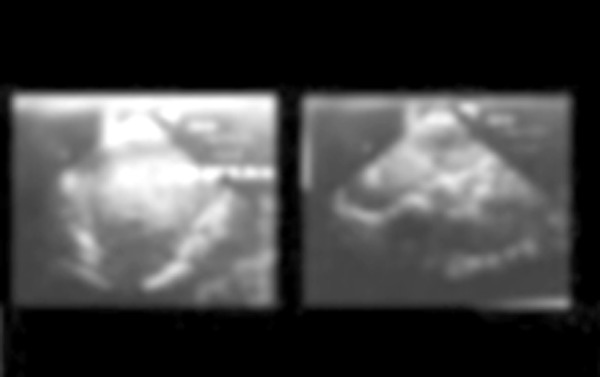
Abdominal sonography

Investigations revealed following results: Abdominal sonography was suggestive of spleenomegaly (16.28 × 8.6 cm), homologous echo texture (Fig. 4). Whereas the size of liver, gallbladder, kidney were normal, no evidence of lymphadenopathy was seen.

The soft tissue attached to malformed tooth was sent for histopathologic examination, which was suggestive of dentigerous cyst (possibility of follicle attached to the partially formed tooth).

The laboratory test revealed Hb 9 mg%, WBC 2.3 × 10^9^/L, DLC showed neutrophils 70% and lymphocytes 30%. Serum chemistry revealed Cl 10^6^ mg/l and alkaline phosphatase 8.0 KA unit/dl.

**Fig. 5:**
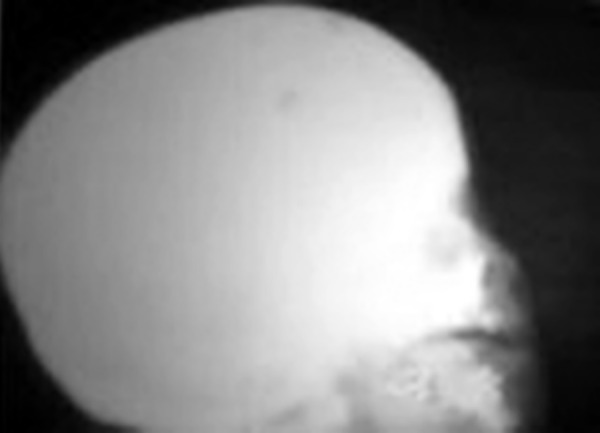
Skull radiograph

**Fig. 6:**
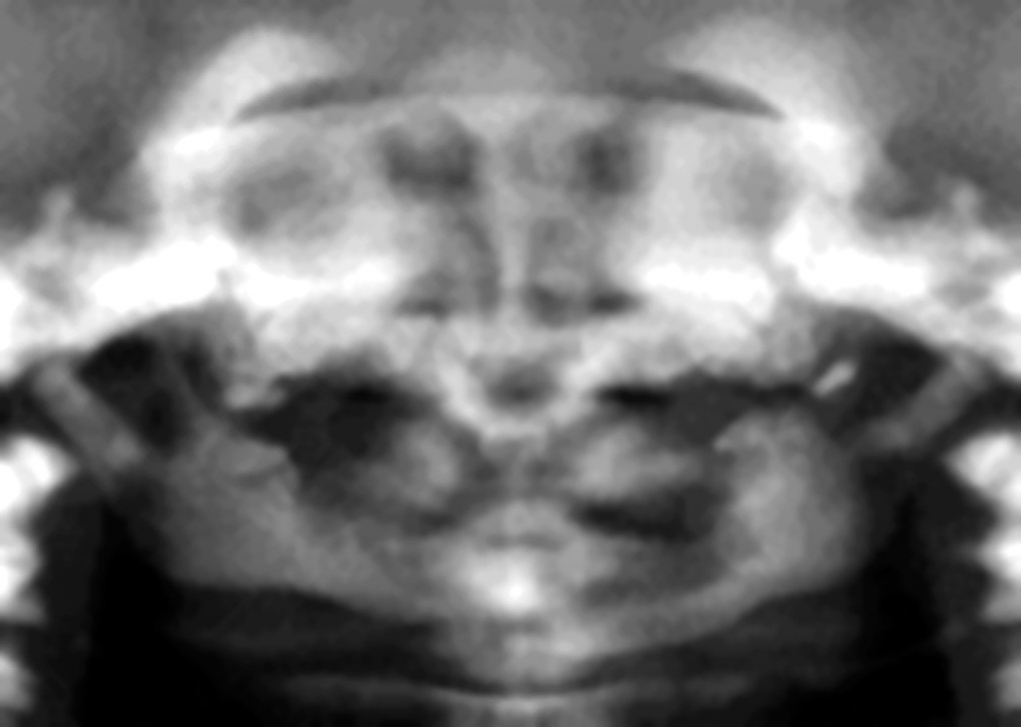
OPG

The skull radiograph and radiograph of left mandible (oblique lateral view) showed increased bone density (Fig. 5). The OPG (Fig. 6) showed that all permanent tooth bud remained totally or partly embedded in basal bone. Vertical growth of alveolar ridge was very limited. Where a fenestration of overlaying mucosa had occurred, a localized, progressive osteitis developed leading to soft tissue inflammation.

## DIFFERENTIAL DIAGNOSIS

It has been documented that classical features of osteopetrosis can be misdiagnosed in cases of Kaffey’s disease, myelofibrosis, skeletal fluorosis, hypoparathyroidism. The confirmatory test would be using brain isoenzyme of creatine kinase, which is a biochemical marker of osteopetrosis.^2^

The patient was diagnosed as a case of osteopetrosis with superadded mandibular osteomyelitis.

## DISCUSSION

Osteopetrosis is a rare metabolic disease characterized by a generalized increase in skeletal mass. Dental abnormalities may be attributed to the pathological changes in osteo-petrosis. Patients with the disease seem to be especially suscept ible to caries. Other dental changes may include delayed eruption and early loss of teeth, enamel hypoplasia, malformed roots and crowns, and thickening of the lamina dura.^5^

The most common complication of the osteopetrosis is pathologic fractures; those with congenital presentation are likely to have most fractures.^6^

Management of the patients with osteopetrosis requires a comprehensive approach to characteristic clinical problems, including hematological and metabolic abnormalities, fractures, deformity back pain, bone pain, osteomyelitis and neurological sequelae.^7^ These patients should receive increased attention on prophylactic dental treatment and oral hygiene maintenance procedures.^2^

**Fig. 7:**
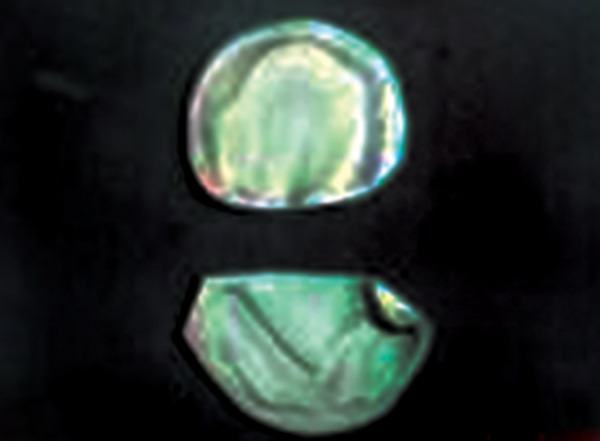
Diagnostic cast

**Fig. 8:**
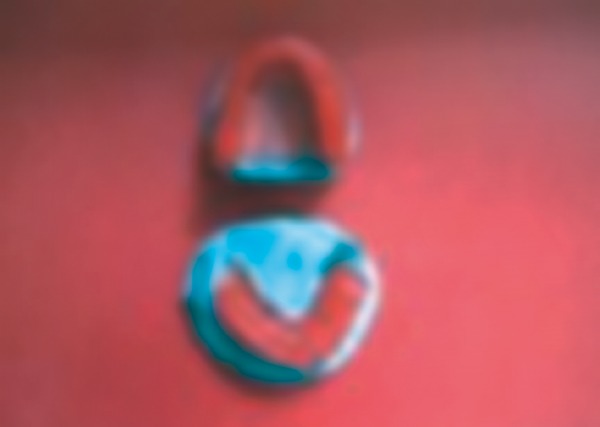
Occlusal rims on master cast

**Fig. 9:**
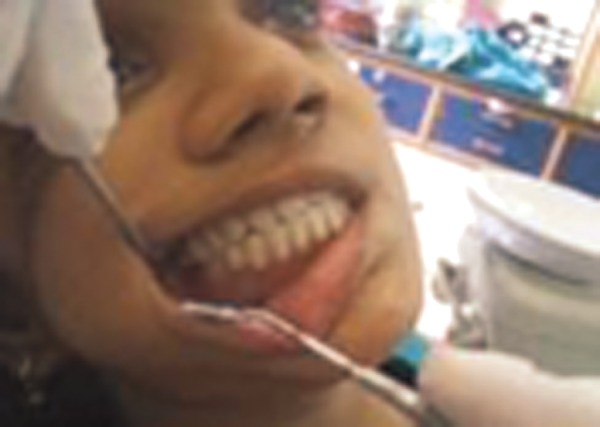
Denture trial in (right side)

**Fig. 10:**
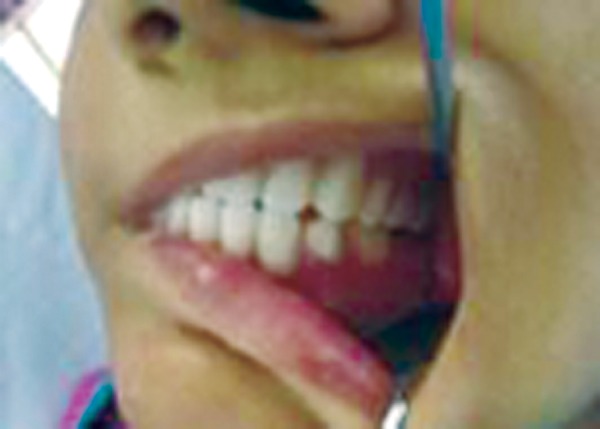
Denture trial in (left side)

**Fig. 11:**
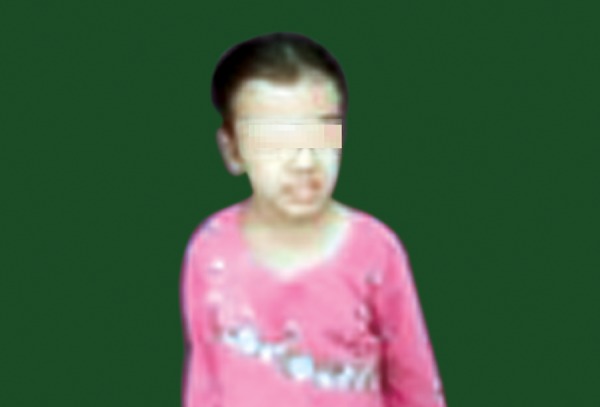
Denture insertion

Complete dentures continue to have an important role in the treatment of edentulous patients. Edentulous ridges though the feature of old age can be seen in pediatric dental patients being affected by certain syndromes, is as seen in this case also. Prior to treatment, diagnostic casts were obtained using irreversible hydrocolloid (putty) (Fig. 7). Custom tray was fabricated using self cure acrylic, which was used later to make secondary/final impression. Conventional border molding procedure with modeling plastic impression compound sticks was done on the fabricated custom trays. After border molding was completed, secondary impression was made with the help of zinc oxide eugenol impression pastes and casts were poured. Occlusal rims were fabricated and jaw relations were recorded in centric relation (Fig. 8). Complete denture is verified for esthetics, phonetics and necessary correction were made (Figs 9 and 10). Denture was delivered and patient was recalled next day for postinsertion (Fig. 11).

Prosthetic treatment includes partial or complete dentures. The maxillary dentures are well retained and well accepted by the children. The mandibular dentures often have poor stability and retention due to mandibular destruction both because of pathology and surgical intervention. In practice only the maxillary dentures are used.^4^

## CONCLUSION

Complete prevention of this complication is not currently possible. Normal growth is a balance between osteoblastic (cells that create new bone tissues) and osteoclastic (cells that destroy old bone tissue) activity. In osteopetrosis, the osteoclasts do not function properly leading to fragile bone status. So, the aim of our therapy should be not only to treat the oral complaints of patient but also to provide a complete physical, mental, psychological rehabilitation of the patient so that he/she may lead a normal healthy life.
